# Guarding Health: A Comprehensive Review of Nosocomial Infections in Sickle Cell Anemia, a Multifaceted Approach to Prevention

**DOI:** 10.7759/cureus.53224

**Published:** 2024-01-30

**Authors:** Nitish Batra, Sourya Acharya, Abhinav Ahuja, Keyur Saboo

**Affiliations:** 1 Medicine, Jawaharlal Nehru Medical College, Datta Meghe Institute of Higher Education & Research, Wardha, IND

**Keywords:** multifaceted approach, vulnerable populations, healthcare challenges, prevention strategies, nosocomial infections, sickle cell anemia

## Abstract

This comprehensive review explores the complex dynamics of nosocomial infections in individuals with sickle cell anemia (SCA) and advocates for a collaborative strategy to enhance prevention. SCA patients, marked by compromised immunity and susceptibility to infections, face unique challenges that necessitate tailored preventive measures. The review underscores the importance of vaccination, antibiotic prophylaxis, education, and environmental hygiene in mitigating the risk of nosocomial infections. Addressing socioeconomic factors, healthcare system limitations, patient-related issues, and cultural considerations is imperative for effective prevention. The call to action emphasizes the pivotal roles of healthcare professionals, policymakers, researchers, and community engagement in implementing targeted interventions. By fostering a collective effort, this review envisions an improved landscape for infection prevention in SCA patients, enhancing their overall health outcomes and quality of life.

## Introduction and background

Sickle cell anemia (SCA) stands as one of the most prevalent genetic disorders, affecting millions of individuals worldwide. This hereditary condition, characterized by a mutated form of hemoglobin, leads to the distortion of red blood cells into a crescent or sickle shape, causing various complications. Patients with SCA often experience recurrent pain episodes, anemia, and organ damage, significantly impacting their quality of life [1]. SCA results from a point mutation in the beta-globin gene, producing abnormal hemoglobin known as hemoglobin S (HbS). When oxygen levels decrease, HbS undergoes polymerization, causing red blood cells to deform and become rigid. This process triggers a cascade of events, including vaso-occlusion, hemolysis, and inflammation, contributing to the characteristic clinical manifestations of SCA [2].

Individuals with SCA face unique healthcare challenges due to their compromised immune function, chronic anemia, and increased susceptibility to infections. Among these challenges, nosocomial infections pose a particularly grave threat [3]. Nosocomial infections, or healthcare-associated infections (HAIs), are acquired during medical treatment within healthcare facilities. In SCA, the impact of nosocomial infections can be severe, exacerbating existing health issues and leading to prolonged hospital stays [4].

This comprehensive review aims to critically examine the intricate relationship between SCA and nosocomial infections, shedding light on the multifaceted aspects of prevention. By delving into the epidemiology, risk factors, and outcomes associated with nosocomial infections in SCA patients, this review aims to thoroughly understand the challenges faced by both patients and healthcare providers. Furthermore, the review will explore existing and potential prevention strategies, emphasizing the need for a holistic and integrated approach to safeguard the health of individuals with SCA.

## Review

Understanding nosocomial infections

Definition and Types of Nosocomial Infections

Nosocomial infections, or HAIs, encompass a broad spectrum of infections that patients acquire while receiving medical care within healthcare facilities. These infections can manifest in various forms, including respiratory tract infections, urinary tract infections, surgical site infections, and bloodstream infections (BSIs). Their onset typically occurs 48 hours or more after admission to a healthcare facility or within 30 days after undergoing a medical procedure [5].

Risk Factors for Nosocomial Infections in SCA Patients

Individuals experiencing an immunocompromised state due to SCA confront heightened infection vulnerability. The chronic anemia inherent in SCA and the frequent occurrence of functional asplenia in affected individuals collectively contribute to a weakened immune response. This perpetual state of immune compromise places such individuals at an elevated risk of contracting infections, exacerbating the intricate health challenges associated with SCA [6]. The relentless nature of SCA necessitates frequent hospitalizations for the management of debilitating pain, required blood transfusions, and various other medical interventions. These recurrent and sometimes prolonged stays within healthcare settings substantially increase the exposure of individuals with SCA to potential sources of nosocomial infections. The cumulative effect of multiple hospital visits accentuates the need for stringent infection prevention strategies to safeguard this already vulnerable patient population [5].

SCA patients routinely undergo invasive medical procedures, including central venous catheter placements and surgical interventions. While essential for managing disease complications, these procedures concurrently create additional entry points for pathogens. The invasive nature of such medical interventions significantly heightens the susceptibility of individuals with SCA to nosocomial infections, emphasizing meticulous attention to infection control measures during and after these procedures to mitigate associated risks [7]. Hydroxyurea, a commonly prescribed pharmacological agent for SCA, provides substantial benefits in mitigating symptoms and reducing complications. However, its immunomodulatory effects may pose additional risks regarding nosocomial infections. Hydroxyurea therapy could inadvertently render individuals more susceptible to opportunistic pathogens encountered in healthcare settings by suppressing the immune system. This underscores the imperative for vigilant monitoring and tailored infection prevention strategies for patients undergoing hydroxyurea treatment to maximize its benefits while minimizing associated risks [8].

The vulnerability of individuals with SCA to nosocomial infections is further nuanced by age and coexisting conditions. Pediatric patients with SCA, owing to the immaturity of their immune systems, face an increased susceptibility to infections, including those acquired in healthcare settings. Additionally, individuals with concurrent health conditions, such as asthma or chronic lung disease, experience heightened risks. The interplay of age-related immune system factors in children and compromised respiratory function in those with coexisting conditions underscores the importance of tailored preventive measures to safeguard these subpopulations within the broader sickle cell community [9].

Impact of Nosocomial Infections on Individuals With SCA

Nosocomial infections pose a significant threat to individuals with SCA, precipitating severe complications, intensifying the clinical course of the disease, and increasing the likelihood of morbidity and mortality. The compromised immune status of these individuals exacerbates the impact of nosocomial infections, highlighting the critical importance of robust infection prevention strategies to mitigate these adverse outcomes [10]. Infections acquired within healthcare settings often lead to prolonged hospital stays for sickle cell patients, disrupting the continuity of their lives and imposing additional emotional and financial burdens on patients and their families. Extended hospitalization strains healthcare resources and underscores the imperative for comprehensive infection control measures to curtail the incidence of nosocomial infections and their associated complications [11].

Nosocomial infections pose a risk to the efficacy of ongoing treatments for SCA, such as blood transfusions or disease-modifying therapies. The presence of infections may interfere with the intended therapeutic outcomes, necessitating adjustments to treatment plans and potentially compromising the overall management of the disease. Vigilant infection prevention measures are essential to safeguard the effectiveness of these critical therapeutic interventions [12]. Pain crises are a hallmark manifestation of SCA, and nosocomial infections introduce additional complexities to pain management strategies. The confluence of infections and pain crises creates a challenging scenario for healthcare providers, making providing adequate relief to patients more arduous. This underscores the importance of a holistic approach to infection prevention, not only for the direct impact on morbidity and mortality but also for its indirect consequences on managing pain and overall patient well-being [13].

Epidemiology of nosocomial infections in SCA

Incidence Rates

Nosocomial infections, or hospital-acquired infections, are a significant concern for patients with SCA. These infections can lead to severe complications and even death [10]. Infections, especially pneumococcal septicemia, meningitis, and *Salmonella* osteomyelitis, are significant causes of morbidity and mortality in patients with sickle cell disease (SCD) [14]. The risk of infectious complications is highest in children with a palpable spleen before six months of age [14]. A study conducted in Cameroon determined the burden and spectrum of bacterial infections among SCD children. The study found that the different types of infection included urinary tract infections (13.5%), myositis (8.3%), arthritis (6.3%), and osteomyelitis (4.2%) [15]. In another study, the incidence rate of BSI in adults with SCD was 1.2 episodes per 100 patient-years [16]. Nosocomial BSI occurred in 34 episodes (49%) [17]. In contrast to BSI in children with SCD, *Streptococcus pneumoniae* was rarely encountered in adults with SCD [17]. A high incidence of staphylococcal BSI in adults with SCD was noted, with 28% of all BSI caused by *Staphylococcus aureus* and 15 out of 22 isolates (68%) being methicillin resistant [17]. In low- and middle-income countries, infections remain the leading cause of overall mortality in patients with SCD due to increased exposure to pathogens, increased comorbidities such as malnutrition, lower vaccination rates, and diminished access to definitive treatment [10]. The epidemiology of nosocomial infections in SCA is multifaceted, with various infections affecting patients of different ages. Preventive measures, such as infection control protocols, vaccinations, and antibiotic prophylaxis, are essential to reduce the risk of these infections in SCA patients [18].

Common Pathogens Involved

Patients with SCA are at increased risk of infections, including nosocomial infections. The common pathogens involved in nosocomial infections in SCA patients include bacteria, viruses, parasites, and mycobacteria [10]. *S. pneumoniae* is a common cause of pneumococcal septicemia and meningitis in SCA patients [14]. *Salmonella* SCA patients are at increased risk of *Salmonella* infections, which can cause osteomyelitis and other serious complications [14]. *S. aureus* is a common cause of BSI in adults with SCD. Among all BSI cases, 28% are attributed to *S. aureus*, and of these, 15 out of 22 isolates (68%) are methicillin resistant [16]. Preventive measures, such as infection control protocols, vaccinations, and antibiotic prophylaxis, are essential to reduce the risk of these infections in SCA patients [4].

Severity and Outcomes of Nosocomial Infections in This Population

Nosocomial infections in patients with SCA can lead to severe outcomes, including prolonged hospital stays and death. A study described eight serious, nosocomially transmitted infections in four adult patients hospitalized for complications of SCD, resulting in one patient’s death and prolonged hospital stays for the remaining three [4]. The cases presented in the study suggest that these patients may be at increased risk of nosocomial infections, and risk can be reduced if healthcare workers are especially vigilant in adhering to handwashing and other infection control measures when caring for these patients [4]. Patients with SCA face increased susceptibility to nosocomial infections due to compromised immune systems, frequent hospitalizations, and increased exposure to pathogens [5,10]. In low- and middle-income countries, infections remain the leading cause of overall mortality in patients with SCA due to increased exposure to pathogens, increased comorbidities such as malnutrition, lower vaccination rates, and diminished access to definitive treatment [10]. Nosocomial infections in patients with SCA can have severe outcomes, and preventive measures, such as strict adherence to infection control measures, are essential to reduce the risk of these infections and their associated complications [4,5,10].

Multifaceted prevention approaches

Hand Hygiene and Infection Control

Importance of hand hygiene in healthcare settings: Reducing microbial transmission is crucial in healthcare settings, where hands are prominent vectors for infectious agents. Proper hand hygiene, whether thorough handwashing with soap and water or alcohol-based hand sanitizers, is pivotal in mitigating this risk. Healthcare providers can significantly diminish the microbial load on their hands, thereby protecting themselves and preventing the inadvertent transfer of pathogens to vulnerable individuals, such as those with SCA. Due to their compromised immune status, individuals with SCA are particularly susceptible to infections, making meticulous hand hygiene an essential component of infection prevention strategies in healthcare settings [19]. Given their compromised immune status, SCA patients are particularly susceptible to infections when protecting vulnerable populations. Rigorous adherence to hand hygiene protocols acts as a protective barrier for healthcare providers and other patients and specifically shields individuals with SCA from potential nosocomial threats. Recognizing the heightened susceptibility of these patients, healthcare professionals can significantly contribute to their well-being by consistently practicing proper hand hygiene. This practice minimizes the risk of introducing harmful pathogens that could lead to severe complications in individuals grappling with SCA [20]. Preventing cross-contamination is of paramount importance in healthcare settings where patients with diverse medical conditions coexist. Thorough hand hygiene practices among healthcare providers create a safer environment, effectively minimizing the risk of introducing infections to susceptible individuals, including those with SCA. The prevention of cross-contamination is not only an individual responsibility but also a collective commitment to the well-being of all patients. Proper hand hygiene emerges as a cornerstone in this collective effort, ensuring a safer healthcare environment and reducing the likelihood of nosocomial infections, particularly in populations with heightened vulnerability [21].

Implementing infection control measures: Appropriate personal protective equipment (PPE) is fundamental to infection prevention for healthcare providers. Gloves, masks, and gowns, tailored to the nature of patient care and the potential for exposure to infectious agents, constitute essential barriers. The consistent and proper use of PPE serves as a critical line of defense, effectively preventing the transfer of pathogens between healthcare personnel, patients, and the surrounding environment. Adherence to PPE protocols is paramount to safeguarding the health of providers and the vulnerable patient population, including those with SCA [22]. Regular and effective cleaning and disinfection of healthcare environments form the cornerstone of infection control strategies. Targeting high-touch surfaces and medical equipment, routine disinfection aims to mitigate the persistence of pathogens, thereby reducing the risk of nosocomial infections. Healthcare facilities can create a safer milieu through meticulous environmental cleaning practices, minimizing potential reservoirs of infectious agents and enhancing overall patient safety [23].

The implementation of isolation precautions represents a targeted strategy for containing the spread of pathogens, particularly when caring for SCA patients with known or suspected infections. Whether employing contact or airborne precautions, strict adherence to isolation protocols is indispensable in preventing the transmission of infectious agents within healthcare facilities. This measure is vital for safeguarding individuals with compromised immune systems, such as SCA [24]. Ongoing education and training programs are indispensable in cultivating a culture of infection control among healthcare personnel, patients, and visitors. These programs encompass a range of topics, including proper hand hygiene techniques, the significance of PPE utilization, and the understanding of when and how to implement isolation precautions. By imparting this knowledge, healthcare facilities empower stakeholders to contribute actively to infection prevention, fostering a collaborative and informed healthcare environment [25]. Establishing robust surveillance systems is imperative for monitoring infection rates, identifying potential outbreaks, and tracking adherence to infection control measures. Continuous surveillance provides real-time data, enabling prompt intervention and the adjustment of strategies to address emerging challenges. By maintaining a vigilant and proactive approach to surveillance and monitoring, healthcare facilities can enhance their responsiveness to infectious threats, ultimately contributing to the prevention of nosocomial infections [26].

Vaccination Strategies

Overview of recommended vaccinations for SCA patients: Pneumococcal vaccines are crucial components of the healthcare regimen for SCA individuals who are highly susceptible to pneumonia. Incorporating both PPSV23 and PCV13, these vaccines protect *S. pneumoniae*, a bacterium known to cause respiratory infections, including pneumonia. By enhancing the immune response against this pathogen, pneumococcal vaccines play a vital role in diminishing the risk and severity of pneumonia in SCA patients, thereby improving overall respiratory health [27]. The *Haemophilus influenzae* type B (Hib) vaccine protects against Hib, a bacterium posing a substantial threat to individuals with SCA. This vaccine is instrumental in preventing severe infections, such as meningitis and pneumonia, which can have detrimental consequences for individuals grappling with SCA. By conferring immunity against Hib, this vaccine significantly contributes to the overall protection and well-being of individuals with this hematological disorder [28].

SCA patients face an elevated risk of meningococcal disease, making meningococcal conjugate vaccines (MenACWY) a crucial component of their preventive healthcare measures. These vaccines offer protection against *Neisseria meningitidis*, the bacterium responsible for meningococcal infections. By strengthening the immune response against this pathogen, meningococcal vaccines help mitigate the risk of severe and potentially life-threatening meningococcal disease in individuals with SCA [29]. In light of the increased susceptibility of SCA patients to respiratory infections, the annual administration of influenza vaccines is essential as a preventive measure. These vaccines are designed to protect against the flu, reducing the likelihood of severe complications. Given the potential for respiratory infections to exacerbate the health challenges of individuals with SCA, timely and regular administration of influenza vaccines is paramount for overall health maintenance [30].

Considering the susceptibility of SCA patients to liver-related complications, vaccination against both hepatitis B and hepatitis A is recommended. These vaccines contribute to the prevention of hepatitis-related complications, safeguarding the liver health of individuals with SCA and ensuring a more robust overall health profile [31]. SCA patients may face a higher risk of severe complications from varicella (chickenpox) and herpes zoster (shingles). Varicella vaccination is, therefore, a crucial preventive measure to mitigate the risks associated with these viral infections. By providing immunity against the varicella-zoster virus, this vaccine plays a pivotal role in reducing the severity of varicella and preventing potential complications in individuals with SCA [32]. In regions where malaria is endemic, SCA patients should receive appropriate antimalarial prophylaxis to prevent complications associated with *Plasmodium falciparum* infection. Malaria prophylaxis is an integral part of the healthcare strategy for individuals with SCA, helping to reduce the risk of malaria-related complications and ensuring a more comprehensive approach to their well-being, particularly in areas where malaria is prevalent [33].

Challenges and solutions in vaccination adherence: Limited access to healthcare services, particularly in economically disadvantaged areas, poses a significant barrier to timely vaccination for individuals with SCA. Improving healthcare infrastructure, including establishing accessible clinics and vaccination centers and implementing outreach programs, is crucial. These efforts aim to bridge the gap in healthcare access, ensuring that individuals, regardless of their economic status, can receive vaccinations promptly and conveniently [34]. Vaccine hesitancy, rooted in reservations or misinformation about vaccines, can hinder vaccination efforts. Addressing this challenge requires comprehensive education campaigns and communication strategies. Providing accurate information about the safety and efficacy of vaccines, dispelling myths, and fostering awareness about the importance of vaccinations are essential components of mitigating hesitancy. Engaging with communities through open dialogue can help build trust and alleviate concerns [35].

The fear of injections and the pain experienced by SCA patients can contribute to vaccine hesitancy. Healthcare providers can adopt patient-centered approaches to address these concerns. This may involve alternative administration methods, such as using numbing creams or smaller needles to minimize discomfort. By tailoring vaccination practices to accommodate the unique needs and sensitivities of individuals with SCA, healthcare providers can enhance vaccine acceptance and adherence [36]. Chronic health issues may divert attention from preventive care, including vaccinations. Integrating vaccination into routine healthcare management plans for individuals with SCA is crucial. Emphasizing the importance of vaccinations as a preventive measure and highlighting their role in maintaining overall health can enhance adherence. This approach ensures vaccinations become integral to comprehensive care for individuals managing chronic health conditions [37]. Cultural beliefs and socioeconomic factors influence healthcare decisions, including vaccine uptake. Tailoring vaccination programs to align with cultural norms and values is essential. Additionally, financial assistance or removing economic barriers can enhance vaccine uptake, particularly among individuals facing socioeconomic challenges. Culturally sensitive education campaigns and community engagement initiatives contribute to a more inclusive and accessible approach to vaccination, addressing the diverse factors that influence healthcare decision-making [38].

Antibiotic Prophylaxis

Role of antibiotics in preventing infections: Prophylactic antibiotics are crucial in reducing the risk of bacterial infections in individuals, particularly those with conditions like SCA, who are highly susceptible to specific pathogens. Given the heightened vulnerability to *S. pneumoniae*, especially in individuals with conditions like SCA, prophylactic antibiotics are essential in minimizing the risk of pneumococcal infections. Commonly prescribed antibiotics for prophylaxis, such as penicillin or amoxicillin, serve as preventive measures against a range of pneumococcal infections, including pneumonia and sepsis. This approach is particularly important for individuals with compromised immune systems [39]. *H. influenzae* infections are also addressed through antibiotic prophylaxis, protecting against respiratory infections like pneumonia and meningitis. Prophylactic antibiotics, in this context, act as a preemptive measure to reduce the risk of *H. influenzae* infections, which can be especially detrimental to individuals with conditions like SCA. This targeted use of antibiotics is a strategic approach to safeguarding the health of vulnerable populations [40]. To prevent meningococcal disease, especially in individuals who have not received the meningococcal vaccine, antibiotics such as ciprofloxacin or rifampin may be prescribed during high-risk periods. This prophylactic antibiotic approach is employed when there is a known exposure to meningococcal bacteria or during outbreaks, aiming to prevent the development of meningococcal infections, which can progress rapidly and have severe consequences. Prophylactic antibiotic use in this context is time-sensitive and aligns with a comprehensive strategy for managing the risk of meningococcal disease [41]. Tailored to the individual patient history and susceptibility, prophylactic antibiotics may also be considered for other bacterial infections. The decision to prescribe prophylactic antibiotics is often based on carefully assessing the patient’s medical history, risk factors, and potential exposure to specific pathogens. This personalized approach ensures that prophylactic antibiotics are judiciously used to prevent bacterial infections in individuals at heightened risk, contributing to overall health and well-being management [42].

Appropriate selection and administration in SCA patients: Penicillin, commonly penicillin V or amoxicillin, is a prevalent choice for prophylaxis against *S. pneumoniae* in individuals with SCA. Initiated in early childhood and maintained until at least five years of age, this preventive measure addresses the heightened susceptibility of young children with SCA to pneumococcal infections. Dosages are carefully adjusted based on age and weight, ensuring optimal efficacy while minimizing the risk of adverse effects. Penicillin prophylaxis is a cornerstone in the comprehensive approach to managing the health of individuals with SCA [43]. Prophylactic antibiotics such as ciprofloxacin or rifampin may be prescribed for individuals with SCA exposed to meningococcal disease. The choice of antibiotic and the duration of prophylaxis depend on factors such as the patient’s age, the specific strain of *N. meningitidis*, and the nature of the exposure. Meningococcal prophylaxis serves as a targeted intervention to prevent the development of meningococcal infections in individuals at heightened risk, aligning with broader strategies for managing infectious threats [44].

Regularly monitoring patients undergoing antibiotic prophylaxis is essential for assessing its efficacy and detecting any adverse effects. Clinicians must remain vigilant for signs of antibiotic resistance, adapting treatment plans to maintain effectiveness. Monitoring also includes routine assessments of the patient’s overall health to ensure that antibiotic prophylaxis aligns with the individual’s specific needs and health status [45]. Patient education is pivotal to the success of antibiotic prophylaxis. It is crucial to ensure that patients and their caregivers understand the rationale behind antibiotic prophylaxis, proper administration techniques, and potential side effects. Comprehensive education enhances patient adherence to prescribed regimens, optimizing the effectiveness of prophylactic measures and promoting the overall health and well-being of individuals with SCA [46]. In regions where specific infections are prevalent, such as malaria, healthcare providers should consider additional prophylactic measures. Antibiotics with antimalarial properties may be part of a broader strategy to mitigate the risk of infections beyond those directly related to SCA. This comprehensive approach reflects a nuanced understanding of the unique healthcare needs of individuals with SCA, tailoring prophylactic measures to their specific challenges and risks [47].

Education and Empowerment

Patient and healthcare provider education on infection prevention: Patient education initiatives should begin with a comprehensive understanding of SCA, delving into its pathophysiology and the inherent risks of increased susceptibility to infections. Healthcare providers are pivotal in conveying this information to patients, fostering awareness of SCA’s unique challenges. Clear communication regarding the specific infection risks associated with SCA is paramount, empowering individuals to actively participate in their healthcare and take proactive measures to mitigate these risks [48]. Education about the importance of vaccinations is crucial for both patients and healthcare providers. A clear and accessible communication strategy should outline the recommended vaccination schedule, potential side effects, and the protective benefits of vaccines. Emphasizing the role of vaccinations in preventing infections, particularly those that pose a significant risk to individuals with SCA, enhances adherence and coverage. A well-informed patient population, supported by healthcare providers, forms a robust defense against vaccine-preventable infections [49].

Patients should be provided with a comprehensive understanding of antibiotic prophylaxis, including the specific antibiotics prescribed, their purpose in preventing infections, and the importance of adherence to prescribed regimens. Healthcare providers must engage in open and transparent discussions with patients, ensuring they comprehend the rationale behind long-term antibiotic use. This includes addressing potential risks and benefits and allowing patients to make informed decisions about their healthcare. Empowering patients with knowledge about antibiotic prophylaxis promotes a collaborative approach to infection prevention [50]. Basic hygiene practices are foundational to infection prevention for individuals with SCA. Both patients and healthcare providers should receive clear guidance on regular handwashing and personal hygiene measures. Education on infection control within healthcare settings is equally crucial, highlighting the significance of maintaining a clean and safe environment to minimize the risk of nosocomial infections. By instilling these hygiene practices, individuals with SCA can take proactive steps to reduce their infection vulnerability, contributing to overall health and well-being [51].

Empowering patients to participate actively in their healthcare: Encouraging a collaborative approach to healthcare decisions is paramount to fostering a sense of ownership and empowerment among patients with SCA. Healthcare providers should actively engage in shared decision-making, involving patients in developing treatment plans and infection prevention strategies. Incorporating the patient’s perspective, preferences, and values ensures that healthcare decisions align with the individual’s unique needs, promoting a more patient-centered and holistic care experience [52]. Providing patients with the knowledge and tools to self-manage their condition is a powerful form of empowerment. This education encompasses a range of aspects, including recognizing early signs of infection, managing pain crises, and understanding when to seek medical attention. Equipping patients with the skills to participate in their care actively enhances their ability to respond to health challenges. It promotes a sense of autonomy and control over their well-being [53].

Regular medical check-ups are crucial in monitoring the health status of individuals with SCA. Empowering patients to prioritize and attend these regular appointments contributes to proactive health maintenance. These check-ups provide opportunities for early intervention, addressing potential issues before they escalate. Encouraging regular engagement with healthcare professionals reinforces the importance of ongoing monitoring and preventive care [13]. Developing and implementing health literacy programs tailored to the specific needs of individuals with SCA is a crucial strategy for empowerment. These programs aim to enhance understanding and facilitate informed decision-making. Delivery methods can include written materials, digital platforms, and interactive sessions. By improving health literacy, patients are better equipped to navigate the complexities of their condition, make informed choices, and actively participate in their healthcare journey [54].

Recognizing the psychosocial aspects of living with a chronic condition like SCA is vital. Providing resources for mental health support, counseling, and peer-to-peer connections empowers patients to navigate the emotional challenges associated with SCA. Psychosocial support complements medical care, fostering resilience and emotional well-being and addressing the holistic needs of individuals with chronic conditions [55]. Empowering patients to advocate for their healthcare needs and those of the broader sickle cell community is a transformative aspect of patient empowerment. This involves education on patient rights, active involvement in support groups, and opportunities for advocacy at both individual and systemic levels. By cultivating advocacy skills, patients contribute to their well-being and the broader efforts to raise awareness, improve healthcare systems, and influence policies that impact the sickle cell community [56].

Environmental Measures

Importance of maintaining a clean and safe healthcare environment: Maintaining a clean healthcare environment is a fundamental strategy for minimizing the presence of pathogens and, consequently, reducing the risk of infections for SCA patients. This preventive measure is particularly crucial in settings where individuals with compromised immune systems, such as those with SCA, receive care. A clean environment creates a safer space, mitigating potential infections and contributing to patients’ overall well-being [5]. Strict adherence to infection control measures is essential for preventing cross-contamination between patients. This practice reduces the likelihood of spreading infectious agents within healthcare facilities, a particularly relevant concern for individuals with SCA conditions. By implementing and enforcing rigorous infection control protocols, healthcare settings can create an environment that prioritizes patient safety and minimizes the risk of nosocomial infections [57].

A clean environment is a foundational element in enhancing overall patient safety, and this is especially important for individuals with SCA. Patients with compromised immune systems are more susceptible to complications from infections, making a clean healthcare environment a critical component of their safety. By minimizing potential sources of contamination and ensuring a hygienic setting, healthcare facilities contribute to the protection and well-being of patients with SCA [58]. A clean, organized healthcare environment delivers effective medical care. Proper sterilization of medical equipment, appropriate handling of medications, and the overall cleanliness of the facility are crucial factors in ensuring the safety and efficacy of treatments. In an environment that prioritizes cleanliness, healthcare professionals can confidently perform their duties knowing that infection risks are minimized, thereby optimizing the quality of care provided to individuals, including those with SCA [59].

Strategies for minimizing environmental transmission of infections: Strict adherence to hand hygiene protocols among healthcare providers is a fundamental measure in preventing the transmission of infections. Regular handwashing with soap and water or using alcohol-based hand sanitizers is imperative to reduce the risk of contaminating patients, surfaces, and medical equipment. This simple yet effective practice is crucial to infection control efforts within healthcare settings [51]. To eliminate potential pathogens, routine and thorough cleaning of all surfaces, patient rooms, and medical equipment using appropriate disinfectants is essential. Environmental services staff should follow established protocols to ensure comprehensive cleaning practices, contributing to a safe and hygienic healthcare environment. Proper cleaning and disinfection play a pivotal role in preventing the persistence and spread of infectious agents [60]. Implementing isolation precautions, such as contact precautions for patients colonized or infected with multidrug-resistant organisms, is a targeted strategy to prevent the spread of infectious agents within healthcare settings. By isolating individuals who pose a higher risk of transmission, healthcare facilities can contain the spread of infections and protect other patients and healthcare providers [24].

Ensuring proper segregation, disposal, and management of biomedical waste is crucial to reducing the risk of environmental contamination. Healthcare facilities should adhere to guidelines for the safe handling and disposal of infectious waste, preventing the unintended spread of pathogens, and maintaining a clean and safe environment [61]. Adequate ventilation and air exchange in healthcare facilities contribute to reducing the concentration of airborne pathogens. Regular maintenance and monitoring of ventilation systems are essential to ensure optimal performance. Properly functioning ventilation systems help create a healthier indoor environment, minimizing the risk of airborne transmission of infections [62]. Ongoing education and training programs for healthcare staff on infection control measures, environmental cleanliness, and protocol adherence are essential to a comprehensive infection prevention strategy. These programs promote a culture of infection prevention within the healthcare environment, ensuring that staff members are well informed and consistently implement best practices [21]. Regular surveillance of HAIs, coupled with environmental audits, is a proactive approach to identifying areas for improvement. Continuous monitoring allows for the timely implementation of corrective measures to enhance infection control. By systematically evaluating and addressing potential weaknesses in infection prevention practices, healthcare facilities can maintain a high standard of safety and quality of care [21].

Challenges and barriers

Socioeconomic Factors

Limited access to healthcare services presents a significant challenge for individuals with lower socioeconomic status, often impeding their ability to access timely and quality healthcare. Barriers may include the need for closer proximity to healthcare facilities, transportation challenges, or the limited availability of healthcare services in economically disadvantaged areas. Consequently, individuals facing these obstacles may encounter delayed interventions and not receive timely medical attention. Such delays can elevate their susceptibility to infections, emphasizing the critical role of timely diagnosis and treatment in preventing the progression of illnesses [63]. Financial constraints further compound the challenges faced by individuals with lower socioeconomic status. The affordability of medications, vaccinations, and preventive measures can become significant barriers, hindering their ability to access and adhere to prescribed treatments and vaccination schedules. The financial burden associated with essential medications or preventive measures may act as a deterrent, leading to suboptimal adherence. This financial constraint not only impacts individual health outcomes but also contributes to an increased risk of nosocomial infections, as individuals may be unable to afford crucial preventive measures [64]. Inadequate health education is another factor affecting individuals in lower socioeconomic groups. Limited health literacy and awareness about the importance of infection prevention measures may be more prevalent in these populations. Individuals with lower health literacy may require a comprehensive understanding of the significance of preventive strategies such as vaccinations, hand hygiene, and other infection control measures. This lack of understanding can contribute to suboptimal healthcare-seeking behaviors, delays in seeking medical attention, and challenges in adhering to preventive strategies. Inadequate health education thus exacerbates the risk of nosocomial infections, emphasizing the importance of fully informing individuals about protecting themselves from infectious threats. Addressing these disparities and promoting overall health outcomes requires a focus on improving health education and raising awareness [65].

Healthcare System Challenges

Resource limitations, encompassing personnel and medical equipment, present a significant challenge to effectively implementing comprehensive infection prevention measures. In healthcare systems facing resource constraints, the availability of trained staff, proper medical equipment, and adequate facilities may be limited. This shortage can impact the ability to maintain rigorous infection control protocols, compromising patient safety, including those with SCA. Adequate resources are essential for providing a safe and hygienic healthcare environment, reducing the risk of nosocomial infections [66]. Inadequate staff training is another critical concern that needs addressing. There is a pressing need for more training and education among healthcare providers regarding the unique needs of SCA patients and best practices for infection prevention. Insufficient knowledge about the specific vulnerabilities of individuals with SCA and the necessary precautions may lead to suboptimal care. Comprehensive training programs addressing the distinct aspects of caring for SCA patients, including infection prevention strategies, are crucial for ensuring that healthcare providers have the knowledge and skills to deliver high-quality care and minimize infection risks [5]. The fragmentation of healthcare delivery is yet another challenge. The fragmented nature of healthcare services and a lack of coordination between different providers and facilities can result in gaps in care. This fragmentation may lead to missed opportunities for preventive interventions and follow-up care for individuals with SCA. The lack of seamless communication and coordination between healthcare providers can contribute to increased risks of nosocomial infections. A more integrated and coordinated approach to healthcare delivery is essential to ensure continuity of care, timely interventions, and effective infection prevention measures for individuals with SCA [67].

Patient-Related Factors

Limited health literacy poses a significant challenge for patients with SCA in terms of understanding and implementing infection prevention strategies. Health literacy encompasses comprehending medical information, following treatment plans, and recognizing early signs of infection. Individuals with SCA, facing limited health literacy, may struggle to fully understand medical instructions, leading to suboptimal adherence to preventive measures and treatment regimens. Improving health literacy through targeted education and clear communication is crucial to empower patients to actively participate in their healthcare and reduce the risk of infection [68]. Chronic pain and fatigue further complicate matters for SCA patients. Dealing with persistent pain and fatigue can significantly impact their ability to engage in preventive measures and adhere to treatment plans. These conditions may limit mobility, making it challenging for patients to implement physical activities that promote overall health and immune function. Pain management strategies, often involving medications that affect energy levels and daily functioning, contribute to an increased risk of infections as patients navigate the complex interplay of managing their chronic symptoms and adhering to infection prevention practices [69]. Psychosocial factors, including anxiety, depression, and stigma, also play a crucial role in a patient’s overall well-being and willingness to engage in infection prevention practices. Individuals with SCA may grapple with the emotional burden of living with a chronic condition, the potential stigma associated with the disease, and the psychological impact of recurrent hospitalizations. These factors can contribute to stress, impacting immune function and the ability to participate proactively in infection prevention. Addressing psychosocial factors through holistic healthcare approaches, including mental health support and counseling, is vital for enhancing overall patient well-being and reducing the risk of nosocomial infections [70].

Cultural Considerations

Traditional beliefs and practices and cultural factors significantly influence healthcare decisions, particularly in SCA. Understanding and respecting cultural nuances is crucial for healthcare providers dealing with SCA patients. Cultural competence involves recognizing and addressing the impact of cultural factors on healthcare choices. By adopting a culturally sensitive approach, healthcare providers can ensure effective communication, collaboration, and the incorporation of traditional beliefs into healthcare plans. This fosters trust and improves the likelihood of adherence to preventive strategies [71]. The stigma associated with SCA and misconceptions about the causes and transmission of the disease can create barriers to open communication between patients and healthcare providers. Stigmatization may lead to a reluctance to seek medical care, share relevant health information, or adhere to preventive measures. Addressing and dispelling misconceptions through patient education and community awareness initiatives are crucial steps in mitigating the impact of stigma. Open and non-judgmental communication from healthcare providers can help build trust and facilitate a supportive healthcare environment [72]. Culturally sensitive community engagement and outreach programs are essential for fostering trust and understanding within diverse communities, including those affected by SCA. Community-specific factors may influence healthcare-seeking behaviors, and engagement initiatives should be tailored to address cultural norms, beliefs, and preferences related to health and healthcare. These programs aim to build strong relationships between healthcare providers and the community, promoting awareness, trust, and collaboration. Through community engagement, healthcare systems can better understand and navigate cultural factors that may impact the successful implementation of infection prevention measures, ultimately improving health outcomes for individuals with SCA [73].

## Conclusions

In conclusion, the intricate relationship between SCA and nosocomial infections demands a comprehensive and collaborative approach from healthcare professionals, policymakers, and researchers. This review has emphasized the importance of multifaceted prevention strategies, including vaccination, antibiotic prophylaxis, education, and environmental measures, to mitigate the heightened risk of infections in SCA patients. Recognizing the challenges posed by socioeconomic factors, healthcare system limitations, patient-related issues, and cultural considerations is crucial for devising targeted interventions. As a call to action, healthcare professionals are urged to prioritize ongoing education and consistent implementation of infection control protocols. Policymakers should allocate resources to improve healthcare infrastructure and support preventive measures, and researchers should delve into innovative strategies. Additionally, community engagement, including awareness campaigns and partnerships with advocacy groups, is essential to address cultural factors influencing healthcare decisions. Through these collective efforts, it is possible to enhance the prevention of nosocomial infections in individuals with SCA, ultimately improving their overall health outcomes and quality of life.
